# Macrophage Inflammatory Protein-1α Shows Predictive Value as a Risk Marker for Subjects and Sites Vulnerable to Bone Loss in a Longitudinal Model of Aggressive Periodontitis

**DOI:** 10.1371/journal.pone.0098541

**Published:** 2014-06-05

**Authors:** Daniel H. Fine, Kenneth Markowitz, Karen Fairlie, Debbie Tischio-Bereski, Javier Ferrandiz, Dipti Godboley, David Furgang, John Gunsolley, Al Best

**Affiliations:** 1 Department of Oral Biology, School of Dental Medicine, Rutgers University, Newark, New Jersey, United States of America; 2 Department of Periodontics, School of Dentistry, Virginia Commonwealth of Virginia, Richmond, Virginia, United States of America; Columbia University, United States of America

## Abstract

Improved diagnostics remains a fundamental goal of biomedical research. This study was designed to assess cytokine biomarkers that could predict bone loss (BL) in localized aggressive periodontitis. 2,058 adolescents were screened. Two groups of 50 periodontally healthy adolescents were enrolled in the longitudinal study. One group had *Aggregatibacter actinomycetemcomitans* (*Aa*), the putative pathogen, while the matched cohort did not. Cytokine levels were assessed in saliva and gingival crevicular fluid (GCF). Participants were sampled, examined, and radiographed every 6 months for 2–3 years. Disease was defined as radiographic evidence of BL. Saliva and GCF was collected at each visit, frozen, and then tested retrospectively after detection of BL. Sixteen subjects with *Aa* developed BL. Saliva from *Aa*-positive and *Aa*-negative healthy subjects was compared to subjects who developed BL. GCF was collected from 16 subjects with BL and from another 38 subjects who remained healthy. GCF from BL sites in the 16 subjects was compared to healthy sites in these same subjects and to healthy sites in subjects who remained healthy. Results showed that cytokines in saliva associated with acute inflammation were elevated in subjects who developed BL (i.e., MIP-1α MIP-1β IL-α, IL**-**1β and IL-8; p<0.01). MIP-1α was elevated 13-fold, 6 months prior to BL. When MIP-1α levels were set at 40 pg/ml, 98% of healthy sites were below that level (Specificity); whereas, 93% of sites with BL were higher (Sensitivity), with comparable Predictive Values of 98%; p<0.0001; 95% C.I. = 42.5–52.7). MIP-1α consistently showed elevated levels as a biomarker for BL in both saliva and GCF, 6 months prior to BL. MIP-1α continues to demonstrate its strong candidacy as a diagnostic biomarker for both subject and site vulnerability to BL.

## Introduction

Improved diagnostics has been a major initiative in all phases of health care for the last 20 years with the anticipation that early diagnosis will lead to effective preventive treatment, reduced medical expenses, and improved overall health [1], [2]. Periodontal research has been in the forefront of these efforts but the search for a biomarker for early prediction of disease has been elusive [3], [4].

Periodontitis affects 47.2% of the adult American population and develops in response to a bacterial challenge; called plaque biofilm [5]. The disease initially affects the gingiva, a complex of tissue forming a collar around the base of the tooth enamel. The locus of infection is a “U” shaped crevice between the gingiva and its adjacent enamel. The gingival crevicular space is lined by epithelium separating the host from its external environment [6]. Periodontal disease is similar to other mucosal infections in that over time the provoking microbial challenge induces an aggressive inflammatory response in the underlying vascular connective tissue [7]. As inflammation progresses the boney structure supporting the tooth in the jawbone is undermined resulting in tooth loosening.

Periodontal researchers have an advantage over other mucosal scientists in that disease progression can be visualized and recorded over a span of time during which minimal irreversible damage occurs. Further, both bacterial initiators and host response elements can be collected from the crevice, the focus of disease activity, in a non-invasive manner [8]. Despite these advantages, progress in periodontal research has been hampered by; 1) difficulties tracking disease progression from beginning to end [9], [10], and 2) difficulties identifying accurate and sensitive markers of disease [11], [12].

Periodontal disease can be divided into two broad categories; a chronic form that occurs primarily in adults, named Chronic Adult Periodontitis (CAP), and an acute form that occurs in adolescents, named Localized Aggressive Periodontitis (LAP) [13]. CAP develops over a non-specified time-period, in an unspecified tooth location. CAP does not restrict itself to specific ethnic groups and is exacerbated by smoking [14]. These factors make CAP difficult to diagnose and follow. In contrast, LAP while less prevalent is relatively easy to diagnose and follow because it is localized to a few specific teeth, is found predominantly in adolescents of African and Hispanic descent, and is highly associated with *Aggregatibacter actinomycetemcomitans* (*Aa*), a Gram-negative microbe purported to be a specific pathogen associated with LAP [6].

The goal of our research has been to identify early markers of LAP, however, the current clinical pathway from health to periodontal disease presents several difficult roadblocks [15], [16]. Standard measures of soft tissue loss, such as probing for pockets and clinical attachment level (CAL) loss, the current standards of diagnostic care, are relatively insensitive [17]. These measurements are particularly poor in LAP because these children are in their mixed dentition stage with teeth continuously erupting [18]. As a result, soft tissue measurements are relatively inaccurate because landmarks are constantly shifting. In contrast, bone measurements are related to specific physiological events that are well studied and occur in alveolar bone that is intimately related to stable tooth support [18]. While these soft-tissue landmarks vary, alveolar bone loss (BL) is irreversible to the greatest extent. However, both soft and hard tissue measurements are downstream relative to disease initiation and represent past historical data.

Notwithstanding these shortcomings identification of bone biomarkers in LAP could provide meaningful information for both forms of periodontal disease because the mechanisms for BL are host related, are common to both diseases (CAP and LAP), and should occur at least 6-months prior to detectable BL by radiograph [19]. Along these lines we have previously identified MIP-1α as a unique biomarker related to bone remodeling in LAP which has recently been confirmed by others in CAP [20]–[22]. However, longitudinal studies encompassing periodontal disease at both the subject and site level have not been done [20]–[22].

This report is part of an ongoing longitudinal cohort study of LAP in adolescents. The overall goals of the study are to identify host and microbial biomarkers of disease development. The purpose of this current study is to identify both subject and site-specific host-related biomarkers that could be predictive of BL in LAP subjects.

## Materials and Methods

### Ethics Statement

Approval for the conduct of this study was obtained from the Institutional Review Board of the University of Medicine and Dentistry of New Jersey (now Rutgers University).

The study was conducted in Newark, New Jersey because the majority of residents in Newark are African American and Hispanic, populations vulnerable to LAP. Both ascent and consent were received from each student’s parent or guardian prior to screening.

### Clinical Procedures

#### Screening

**Figure 1:**
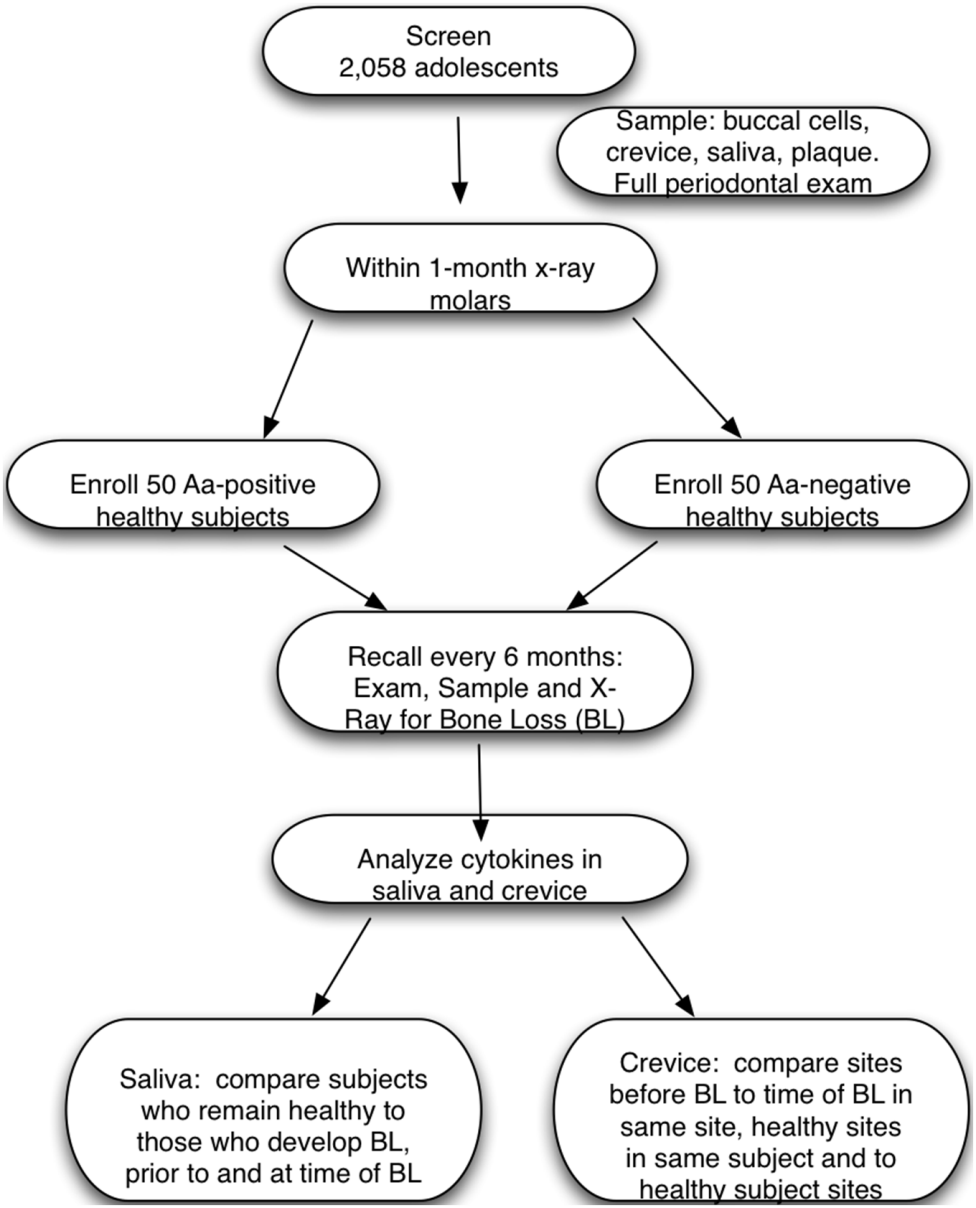
Flow Diagram of subjects participation in study. Fifty *Aa*-positive and 50 *Aa*-negative subjects were enrolled and followed every 6 months for up to 3 years. Cytokines from saliva and crevice fluid were collected every 6 months, stored, and then after bone loss was detected, salivary samples from 10 of those subjects (from a total of 16) and saliva from another 60 subjects who remained healthy was analyzed. In the case of crevice fluid, samples from 16 subjects who developed bone loss and another 38 who remained healthy was available for analysis.

Screening was performed to select a group of healthy *Aa*-positive and *Aa*-negative subjects for enrollment into a longitudinal study designed to assess the association of *Aa*-carriage to host and microbial factors related to development of LAP. The examination consisted of a medical and dental history assessment, an oral examination, and collection of samples of saliva and buccal epithelial cells (BECs). Subjects were included if they were between the ages of 11–16, were medically healthy, and had a minimum of 4 first molars and 10 occluding teeth. Subjects were excluded if they had any medically related issues including but not limited to bleeding disorders, immune deficiencies, were taking either antibiotics or anti-inflammatories, or, if they had extensive dental caries [23]. A flow diagram illustrates the screened and enrolled population included in the study (Fig. 1).

#### Determination of Aa carriage

Buccal cells were used to determine whether the subject was *Aa*-positive or *Aa-*negative as described previously [23]. Briefly, BEC samples were obtained by scraping the cheek with a wooden tongue depressor using 5 strokes to obtain a sufficient amount of buccal material to analyze. The collection was suspended in 1 ml of phosphate buffered saline (PBS). A 100 µl aliquot of the re-suspended BEC sample was plated for determination of the presence or absence of *Aa*. Streaking, dilution and plating was done on AAGM agar for enumeration of *Aa* [23]. After plating on AAGM agar, culture dishes were placed in an incubator at 37°C with 10% CO_2_ for 3–4 days [23]. Colonies seen on plates were identified as *Aa* based on their unique morphology and catalase activity. The polymerase chain reaction (PCR) was used for confirmation of cultural identification [23]. DNA obtained by the DNeasy tissue kit (Qiagen, Inc Valencia CA) for Gram-negative bacteria was used for this assessment [23]. Initially, categorization of subjects as *Aa-*positive was determined by the growth of *Aa* on agar. If no growth occurred on AAGM agar, DNA was extracted from the initial 1 ml BEC sample using the Gram-negative protocol described above for further proof that *Aa* was absent. As previously described DNA primers for the leukotoxin promoter region that is unique to *Aa* was used for these PCR determinations. This procedure was done a minimum of two times to confirm the presence or absence of *Aa* [23]. BECs were obtained from subjects at each visit to confirm their *Aa* status. To be considered to be *Aa-*negative no BEC or pocket sample could be *Aa-*positive. To be considered to be *Aa-*positive, subjects were required to show *Aa-*positivity for BECs or pocket samples at each visit.

### Recall Visits

#### Periodontal examination

Our goal was to enroll at least 50 *Aa*-positive and 50 *Aa*-negative periodontally healthy adolescents and then to follow them and assess their periodontium for BL. Previous data indicated that 25% of *Aa*-positive adolescents develop BL over a 2–3 year period [23]. Probing was performed on six surfaces of all teeth with a Michigan 0 probe. Any pocket 5 mm or greater was examined for clinical attachment loss (CAL). The Loe-Brown definition of incipient disease was used to define subjects as “diseased” (pockets of ≥6 mm and CAL of ≥2 mm) for exclusion from the longitudinal study. Health was defined as pockets of 4 mm or less with no bleeding. A subject with one 5 mm pocket with no bleeding was also considered as healthy [23].

One month following the screening visit, all subjects were required to take four bite-wing radiographs. For purposes of the study, definitive diagnosis of disease was based on x-ray evidence of bone loss as opposed to soft tissue measurements. This decision was based on evidence that soft tissue measurement in this mixed dentition age group could produce shifting landmarks while radiographs provide a more stable diagnosis [23]. Bite-wing x-rays were taken for all subjects at 6-month intervals. BL was indicated by detection of loss of the lamina dura at the alveolar crest. Frequent calibration exercises were conducted for pocket probing, CAL, and radiographic interpretation. The two clinical examiners (KM and DTB) repeated the exercises to determine the accuracy of their readings. For soft tissue, examiners were considered to be in agreement if 80% of the sites they measured had identical readings and if 20% were within ±1 mm. The calibration exercise demonstrated 80% inter and 90% intra examiner repeatability. For x-ray calibration, 95% inter and intra examiner agreement was required.

Only periodontally healthy subjects were enrolled in the study and recalled at 6-month intervals. Samples of saliva, BECs, gingival crevicular fluid (GCF) and subgingival plaque were taken at each visit, given a coded number and stored for future analysis. To avoid disease progression, any subject who showed BL at any visit was exited from the study and provided with treatment at the dental school at no cost. A previous manuscript provides a more detailed description of the clinical procedures [23].

### Sample Collection: Saliva

Students expectorated into a 50 ml wide mouthed polystyrene tube held over ice until approximately 5 ml of unstimulated whole saliva was collected. The salivary sample received a coded number and was subjected to centrifugation at 10,000-×g for clarification and then aliquoted prior to storage at −80°C [23]. After BL was detected samples from that visit and the previous visit were thawed for analysis and comparison [20]. Total protein content and cytokine analysis was performed. Saliva from a total of 16 subjects with BL was acquired; however, in 7 samples a limited amount of saliva was available due to prior analysis [20]. As a result we performed a sample size calculation to determine the number of subjects per group required for further analysis. For that calculation we used salivary IL-1β (as opposed to the MIP-1α data) since this data provided us with a more conservative estimate. Based on these calculations we determined that 9 subjects per group would be sufficient to find a statistically significant differential and to achieve a p value of 0.05. Using these calculations we randomly selected 30 *Aa*-positive subjects and 30 *Aa*-negative subjects who remained healthy to be compared to 10 of the 16 subjects who had LAP. As mentioned, duplicate testing with sufficient saliva was available for 10 subjects in the BL group. Saliva from the remaining 6 BL subjects was depleted and not available for these analyses. Saliva from another 60 healthy subjects who remained healthy was matched to the age and ethnicity of the 10 BL subjects (Table 1).

**Table 1:** Demographic characteristics of subset of subjects for whom salivary cytokine analysis was performed.

Subject Ages (Mean ± Std Dev.)						
Race	Healthy Aa + Subjects (N = 30)		Healthy Aa − Subjects (N = 30)		Bone Loss Subjects (N = 10)	
	Age ± S.D.(Female)	Age ± S.D.(Male)	Age ± S.D.(Female)	Age ± S.D.(Male)	Age ± S.D.(Female)	Age ± S.D.(Male)
Hispanic (N = 17)	12.18±0.83 (6)	14.37±2.11 (5)	16.5±0.00 (3)	14.09±2.19 (5)	16.00±0.00 (1)	12.67±1.47 (2)
African-American (N = 31)	13.61±2.19 (10)	14.09±2.52 (6)	13.5±1.60 (10)	13.71±1.61 (12)	12.35±0.30 (3)	13.23±1.82 (4)
Asian (N = 3)	12.80±0.42 (2)	11.20±0.00 (1)	NA	NA	NA	NA
**Total (N = 51)**	**13.31±2.02 (30)**		**14.03±1.02 (30)**		**13.03±1.53 (10)**	

### Sample Collection: Gingival Crevicular Fluid (GCF)

GCF collection was obtained from the mesial surface of each first molar at each visit. Each quadrant was isolated with cotton rolls and a saliva ejector. Teeth being sampled were gently debrided of supragingival plaque prior to placement of a periodontal paper crevicular collection strip (Oraflow Inc, Plainview NY USA). The collection strip was gently placed at the gingival margin of the molar collection site for 15 seconds. Individual strip samples were placed into coded eppendorf tubes for storage at −80°C [23]. After BL occurred, samples from the BL detection visit and the visit prior to BL were both thawed, eluted in phosphate buffered saline (PBS), and analyzed for protein content and cytokine levels.

For site-specific data we analyzed samples from 15 of the 16 subjects who developed BL. In two subjects, two sites showed BL. We recovered GCF from 17 sites in the 16 subjects who developed BL. For site related data three analyses were performed. First, we analyzed sites that developed BL. GCF was recovered from 15 sites (2 samples were unavailable prior to BL) and assessed those sites at the visit prior to BL (N = 15) as compared to these same sites at the visit BL was detected (N = 17). Second, we analyzed sites that remained healthy in these same 15 subjects (N = 45; the three healthy molars) and compared these sites to sites that developed BL (N = 15). In this case we examined all sites (three other first molar sites that remained healthy in these same subjects) at the visit prior to BL. The LAP samples from sites prior to BL were duplicate samples from the same sites in the previous analysis but were run in a blind manner and independently in a second Luminex analysis (see below). Third, we compared healthy sites in LAP subjects (N = 15) to healthy sites in subjects who did not develop BL (N = 26 for *Aa*-negative subjects and N = 12 for *Aa*-positive subjects). In this case, sample analysis was performed on samples obtained at the visit at the time BL was detected. Prior to selection of samples in this third assessment, we performed a sample size calculation using IL-6 data (as opposed to MIP-1α data). We assumed that IL-6 data would provide the most conservative estimate for sample size calculation. Our analysis indicated that 8 subjects per group would be sufficient to determine significance at the 0.05 level. These calculations indicated that we had sufficient material to proceed.

Subject samples were selected for analysis if there was a sufficient crevicular protein content for analysis and if the subjects were a match to the mean age and ethnicity of the BL subjects. As a result we randomly chose 26 *Aa*-negative subjects (N = 26 sites), 12 *Aa*-positive subjects (N = 12 sites) and 15 healthy sites from the 16 *Aa*-positive subjects who developed BL. All data was coded and analyzed in a blind manner.

For GCF analysis we selected a panel of seven cytokines. IL-1β, MIP-1α, and IL-8 were chosen as markers of acute inflammation. IL-2, IL-10 and IL-12 were chosen as markers of chronic inflammation [24]. We also selected IL-6, a cytokine that participates in both acute and chronic inflammation.

### Cytokine Processing and Analysis

#### Saliva and GCF processing

For salivary analysis the presence and level of 21 chemo/cytokines were assessed using the Luminex/Millipore xMap system (Millipore, Billerica MA) [20]. A 100 µl sample was placed in the well of a 96-well plate containing its own internal controls for each of the chemo/cytokines to be analyzed. Excitation of each fluorochrome produced a signal that permitted detection of from 1–500 pg per chemo/cytokine [20]. All data was normalized to pg/ml based on a constant level of protein. For GCF analysis the presence and level of 7 chemo/cytokines were assessed as described. All test were done twice in duplicate.

### Statistical Analysis

Chi-squared analysis was used to assess differences in race while a student’s-t-test was used to analyze age to determine if the BL subjects differed from healthy controls with respect to age.

Salivary samples were assessed by analysis of variance (ANOVA) comparing 3 groups; samples from healthy subjects who remained healthy (N = 60), salivary samples at the visit prior to BL in healthy subjects who developed BL (N = 10), and samples from these same subjects at the visit BL was detected (N = 10). Tukey Kramer (HSD) analysis was performed to assess all means in pairwise comparisons within the ANOVA. Differences were significant if they achieved a p value of <0.05.

GCF data was subjected to three separate analyses. Initially, sites prior to BL (N = 15) were compared to that same site at the visit BL was detected (N = 17) using an ANOVA with Tukey Kramer analysis. In these same LAP subjects, BL sites in the visit prior to BL were compared to first molar sites that were diagnosed as healthy and remained healthy in these same subjects at that same visit. In this the second analysis, 45 healthy sites (three healthy first molar sites in 15 subjects) were compared to 15 BL sites prior to BL. The groups used for the third analysis were selected based on the sample size calculations derived from the second GCF analysis and were matched in age, race, and availability of GCF for analysis. This material was taken from healthy sites at the time of the BL detection visit from LAP subjects and compared to *Aa*-positive and *Aa*-negative subjects who started healthy and remained healthy. All data was analyzed by ANOVA with Tukey Kramer analysis coupled with a Bonferroni correction to protect against multiple testing (p value = 0.007 was set to achieve a level of significance of p<0.05). Sensitivity and specificity testing was performed using a cut point of >40 pg/ml for MIP-1α. We also calculated positive and negative predictive values using the same sample groups.

Specificity was calculated for another 26 subjects who were *Aa*-negative and remained healthy and another 12 subjects who were *Aa*-positive and remained healthy as compared to the 15 subjects who developed BL. In this analysis a cut point of MIP-1α levels of 40 pg/ml was used for GCF at the visit prior to BL. Specificity was determined by calculating the number of healthy sites from these various subject groups showing MIP-1α levels >40 pg/ml. The rationale for determining the cut-off value for MIP-1α based on the fact that the range for MIP-1α was from 42.5 pg/ml to 52.7 pg/ml with a mean of 47.66 pg/ml and standard deviation of 8.82 pg/ml in sites that encountered BL at the visit prior to BL. A slightly lower level of 40 pg/ml was chosen for the cut point.

Finally, we constructed a statistical model analyzing both the sites (N = 15 BL sites from LAP subject +45 healthy sites from same LAP subject = 60) and the subjects (N = 15 BL subjects +12 healthy *Aa*-positive subjects +26 healthy *Aa*-negative subjects; N = 53) relative to levels of MIP-1α to determine whether the subject or the site was the most critical factor relative to levels of MIP-1α and its relationship to BL. In the analysis the subject was modeled as a random effect.

## Results

### Demographics

The demographic distribution of all students who participated in the study is shown in Tables 1 and 2. Salivary analysis compared 60 healthy subjects to 10 subjects who developed LAP. Fifty percent of the subjects were female with a mean age of 13.03+2 as compared to 13.7+2.3 for the male subjects (Table 1). The LAP group had a mean age of 13.03±1.5. Fifty percent of the healthy subjects were *Aa*-positive while 100% of the LAP subjects were *Aa*-positive. GCF analysis included 15 participants who developed BL in addition to another 26 healthy *Aa*-negative and 12 healthy *Aa*-positive participants. The age of the healthy subjects (13.38±1.9) was not significantly different from the LAP group (14.5±1.5) nor was the ethnic distribution of these participants (Table 2). However, while 20% of the healthy group was *Aa-*positive, 100% of the LAP group had *Aa* (Table 2).

**Table 2:** Demographic characteristics of subset of subjects studied for whom gingival crevicular fluid cytokine analysis on a site specific basis was performed.

Race	Subjects Remaining Healthy (N = 38)	Percent Aa Positive	Bone Loss Subjects (N = 15)	Percent Aa Positive
	Age ± S.D.		Age ± S.D.	
Hispanic	**13.47±1.95** (N = 15)	13%	**14.0±1.5** (N = 5)	**100%**
African-American	**13.3±1.52** (N = 20)	25%	**14.5±2.0** (N = 10)	100%
Not Reporting	**12.75±0.9** (N = 3)	33%		

### Cytokine Analysis

#### Salivary cytokines

**Figure 2:**
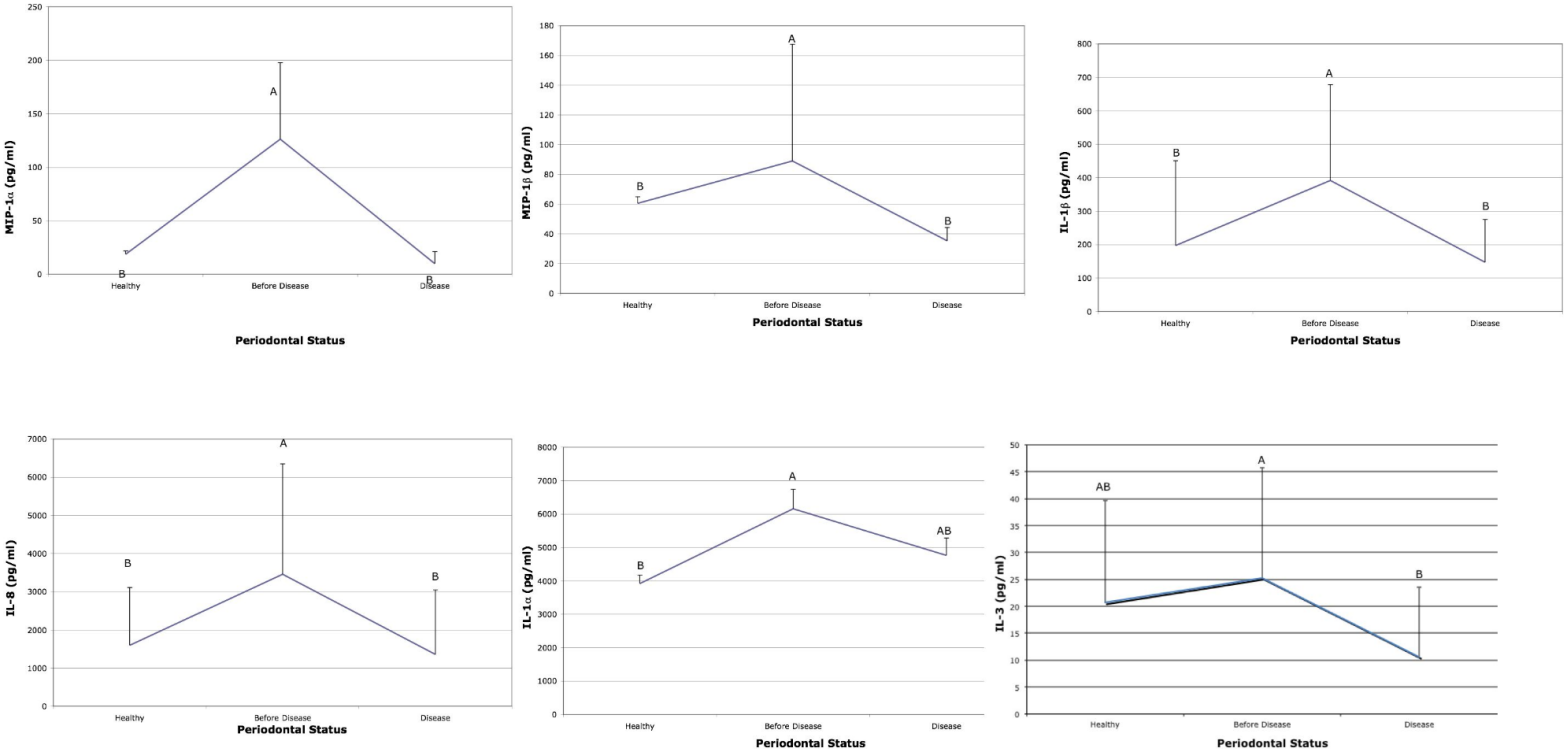
Salivary cytokines from healthy and bone loss subjects: Cytokines elevated prior to bone loss. Saliva from healthy subjects was compared to subjects who developed bone loss. The cytokines that showed significant differences are illustrated 6 months prior to bone loss and compared to levels at the time bone loss was detected (labeled disease) and to salivary levels found in subjects who started healthy and remained healthy. Letters that are different (A vs B) are significantly different at the p<0.05 level. Cytokines MIP-1α, MIP-1β, IL-1β, and IL-8 are all significantly elevated 6-mo. before BL when compared to saliva from healthy subjects and to saliva at the time disease was detected. IL-1α is significantly elevated prior to BL as compared to saliva from healthy subjects but is not significantly different than that seen at the time of disease detection.

**Figure 3:**
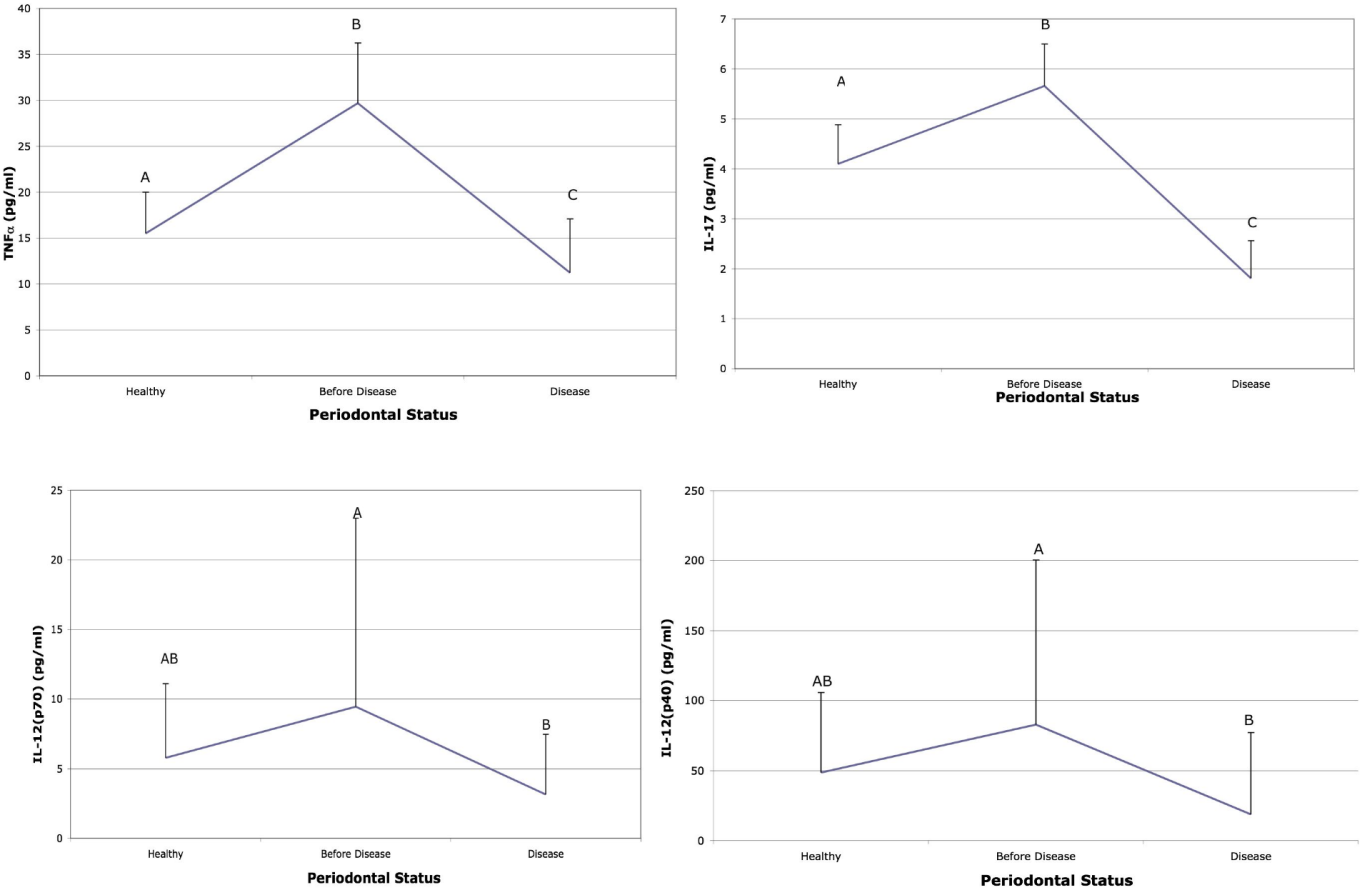
Salivary cytokines from healthy and bone loss subjects: Other salivary cytokines elevated prior to disease. Saliva from healthy subjects was compared to subjects who developed bone loss. The cytokines that showed significant differences are illustrated 6 months prior to bone loss and compared to levels at the time bone loss was detected (labeled disease) and to salivary levels found in subjects who started healthy and remained healthy. Letters that are different (A vs B) are significantly different at the p<0.05 level. Cytokines IL-17 and TNF-α are significantly elevated prior to disease as compared to health and at the time of BL detection; while IL-12 (p40) and IL-12 (p70) were significantly elevated prior to disease but were not significantly different from saliva obtained from healthy subjects p<0.05.

**Figure 4:**
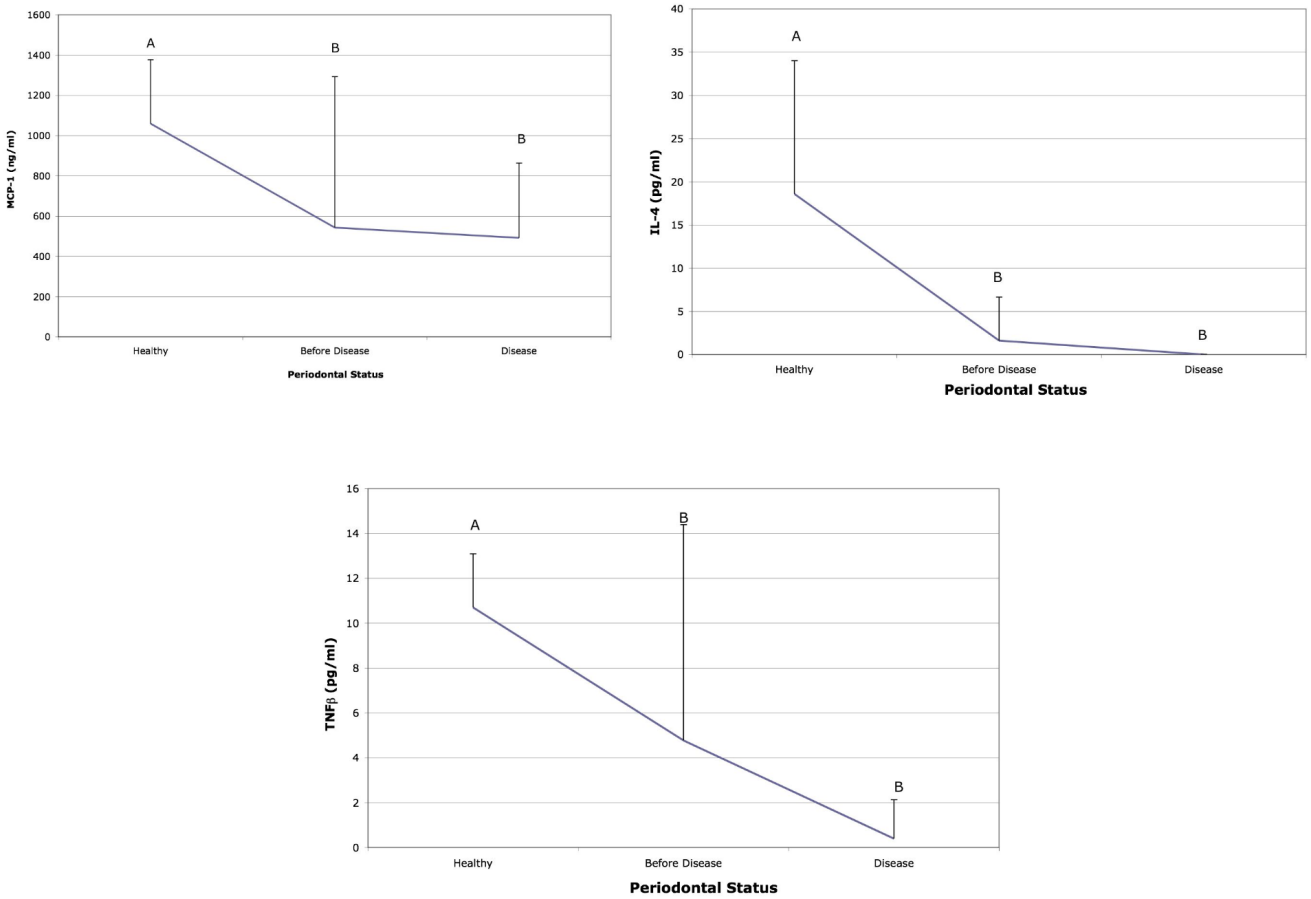
Salivary cytokines from healthy and bone loss subjects: Salivary cytokines elevated in health prior to disease detection. Saliva from healthy subjects was compared to subjects who developed bone loss. The cytokines that showed significant differences are illustrated 6 months prior to bone loss and compared to levels at the time bone loss was detected (labeled disease) and to salivary levels found in subjects who started healthy and remained healthy. Letters that are different (A vs B) are significantly different at the p<0.05 level. Cytokine MCP-1, IL-4 and TNF-β were significantly depressed before disease as compared to health but were not different when saliva prior to disease was compared to saliva at the visit disease was detected.

Cytokine levels associated with acute inflammation (IL-1β, IL-8, MIP-1α and MIP-1β) were elevated in subjects 6–9 months prior to detection of BL as compared to salivary levels at the time BL was detected and to saliva from subjects who remained healthy (Fig. 2). IL-3 and IL-1α were elevated prior to disease when compared to health but showed no difference when compared to the visit when BL was detected (Fig. 2). TNF-α and IL-17 were also elevated prior to BL and at the visit when BL was detected (Fig. 3). In addition, IL-12 was significantly elevated prior to BL in these same LAP subjects but was not elevated in comparison to subjects who remained healthy (Fig. 3). Moreover, IL-4, TNF-β, MCP-1 were all higher in saliva derived from healthy subjects as compared to LAP subjects (Fig. 4). Further, MIP-1α showed the greatest difference when compared to other cytokines and was elevated 13-fold in saliva prior to BL as compared to saliva from healthy subjects and to saliva at the visit BL was detected (Fig. 2). In addition, IL-2, IL-6, IL-7 IL-10 and INF-γ were similar in health and in the visit prior to detection of BL but where lower at the time of detection of BL (Fig. S1).

#### Crevice fluid cytokines

For GCF analysis, MIP-1α was significantly elevated prior to BL at the site of BL (N = 15) as compared to that same site (N = 17) at the time BL was detected (Table 3). In contrast, IL-6 was significantly reduced in sites that developed BL as compared to those sites that remained healthy (N = 45) in these same LAP subjects (N = 15) that developed BL. These assessments were performed on samples at the visit prior to detection of BL (Table 4). MIP-1α levels in GCF taken from these sites (N = 15) in the visit 6–9 months prior to BL showed elevated levels when compared to sites that remained healthy (N = 45) in these same subjects (Table 4; p<0.01).

**Table 3:** Cytokine levels (pg/ml) in same molar sites prior to bone loss vs. same molar sites at visit bone loss detected.

Cytokine levels in pg/ml							
Site Status	IL-1β	1L-2	IL-6	IL-8	IL-10	IL-12	MIP-1α*
Prior to Bone LossN = 15	6.31±11.32	0.93±1.44	0.19±0.22	233±172	1.56±1.36	5.25±6.68	47.66±8.83
Time of Bone LossN = 17	40.07±92.0	1.64±1.60	1.90±1.45	349±234	2.33±1.47	6.68±5.37	21.1±13.3

*MIP-1α levels are higher in crevice fluid obtained from sites prior to bone loss.

**Table 4:** Cytokine levels (pg/ml) in sites remaining healthy vs sites that develop bone loss in LAP subjects: visit prior to detection of BL.

Cytokine levels in pg/ml							
Site Status in LAP subjects(N = 15)	IL-1β	1L-2	IL-6*	IL-8	IL-10	IL-12	MIP-1α**
Sites Remain Healthy(N = 45)	25.9±60.3	1.10±1.48	1.05±1.47	386±445	2.55±1.46	4.72±3.83	14.79±11.04
Sites Develop Bone Loss(N = 15)	6.08±11.03	0.87±1.46	0.18±0.22	218±176.2	1.46±1.33	5.11±6.46	48.03±8.62

*IL-6 levels are higher in crevice fluid in sites that started healthy and remained healthy.

**MIP-1a levels are higher in crevice fluid obtained from sites piror to bone loss that developed bone loss.

In data taken from the third GCF analysis, IL-2 was lower in *Aa-*positive healthy sites as compared to *Aa-*negative healthy sites but was significantly higher in *Aa*-positive healthy sites from LAP subjects (p<0.01). IL-8 and IL-1β were significantly higher in *Aa-*positive healthy sites as compared to *Aa-*negative healthy sites (p<0.01). IL-8 was also elevated in healthy *Aa-*positive sites from LAP subjects (p<0.01; see Table 5).

**Table 5:** Cytokine levels (pg/ml) in healthy sites from LAP subjects vs Aa-negative and positive healthy subjects at visit BL was detected.

Cytokine levels in pg/ml							
Site Status	IL-1β**	1L-2*	IL-6	IL-8***	IL-10	IL-12	MIP-1α*
Healthy Aa-neg (N = 26)	0.88±1.41	2.86±0.25	0.14±0.21	34.3±62.2	2.87±0.93	5.57±4.12	9.39±9.32
Healthy Aa-pos (N = 12)	**59.6**±**115.7**	**0.09**±**0.38**	1.47±4.91	138.8±10.5	1.04±0.65	3.28±2.90	11.04±8.22
LAP (Aa-pos) (N = 15)	26.85±69.8	***4.37***±***1.56***	0.94±1.84	**293**±**373**	2.55±3.90	15.5±4.60	15.5±11.38

*IL-2 higher in sites in LAP subjects vs. Aa-pos & Aa-neg healthy sites.

**IL-1β higher in Aa-pos healthy sites vs. Aa-negative healthy sites.

***IL-8 higher in LAP sites vs. Aa-negative healthy sites.

When a cut point was set at >40 pg/ml and healthy sites in LAP subjects (N = 45) were compared to sites prior to BL (N = 15) in LAP subjects, a specificity of 98.73% and a sensitivity of 93.33% was achieved as a predictive risk marker for MIP-1α in a site prior to BL that would develop BL (Table 6).

**Table 6:** Predictive value of crevicular levels of MIP-1α of 40 pg/ml or above at sites that do or do not develop bone loss in same LAP subject at visit prior to bone loss.

MIP-1α levels	Bone LossPositive Sites(N)	Bone LossNegative Sites(N)	Sensitivity (%)	Specficity (%)	Postiive PredictiveValue	Negative PredictiveValue
MIP-1α >40 pg/ml	14	1				
MIP-1α <40 pg/ml	1	44				
			93.33	98.73	93.3	98.7
TOTAL SITES	15	45				

When 26 subjects who were *Aa*-negative and 12 subjects who were *Aa*-positive all of whom remained healthy were compared to 15 sites that remained healthy in LAP subjects, a specificity of 98% was obtained when the cut point was set at >40 pg/ml for MIP-1α (Table 7). Our statistical modeling indicated that the site status played the more dominant role than subject status relative to prediction of BL (p<0.0001).

**Table 7:** Analysis of specificity of MIP-1a levels in healthy sites from Aa-negative and Aa-positive subjects who remained healthy as compared to healthy sites in LAP subjects where another site developed bone loss.

MIP-1α levels	Healthy site fromAa-negative subject(N = 26)	Healthy site fromAa-positive subject(N = 12)	Healthy site fromLAP subject(N = 15)	Total Sites withMIP-1α levels > or < than40 pg/ml
MIP-1α >40 pg/ml (N)	1	0	0	1
MIP-1α <40 pg/ml (N)	25	12	15	52
TOTALS ALL SITES	26	12	15	53

## Discussion/Conclusions

Our results indicate that elevated levels of MIP-1α in saliva can be used to identify subjects susceptible to BL, while MIP-1α in GCF can identify sites susceptible to BL. We followed adolescents vulnerable to LAP from health to disease over a 2–3 year period and found that elevated levels of MIP-1α were seen in advance of BL at both the site and subject level. These elevated levels were detected 6–9 months prior to radiographic visualization of BL. The relationship between MIP-1α and periodontal disease has been confirmed in two independent studies [21], [22]. One study showed that MIP-1α was a superior salivary biomarker when compared to further downstream markers of BL that included osteoprotegrin (OPG), C-telopeptide pyrodiniline cross links of type 1 collagen, and a β-terminal C-type 1 collagen telopeptide [22]. MIP-1α showed a specificity of 92.7% and a sensitivity of 94% for periodontal disease and an 18-fold elevation in CAP subjects when their salivary biomarkers for bone remodeling were compared to healthy subjects. It was particularly revealing that MIP-1α (as an upstream mediator of bone loss) performed significantly better than mid (OPG) and downstream (telopeptides) markers of bone remodeling [22]. In a second study, statistically elevated levels of MIP-1α were found in GCF obtained from diseased pocket sites in LAP [21]. While both studies support the utility of MIP-1α as a biomarker, neither presented longitudinal data implicating MIP-1α as a predictor of BL and neither examined both salivary and crevicular biomarkers in the same subject.

Microbial profiles appear to be distinctly different when comparing acute (LAP) and chronic (CAP) forms of periodontitis [23]. This is reflected by evidence indicating that events related to Toll-like receptors expressed by the innate immune pattern recognition system are also distinctly different when it comes to differentiating between bacteria related to CAP and LAP [24]. However, as events move further and further downstream toward RANK and MIP-1α activation of osteoclasts, these events appear to flow into a common pathway [19], [23]. Therefore, the data derived from this study is likely to be applicable to BL in CAP [22].

MIP-1α is chemotactic for polymorphonuclear leukocytes (PMNs) in acute inflammation and is stimulatory for monocytes in relation to osteoclastogenesis [25]. In vitro it has been shown that *Aa* lipopolysaccharide (LPS) can induce PMNs and epithelial cells to produce MIP-1α. MIP-1α can activate osteoclasts, which can also be synergized by IL-1β [25]. This relationship between *Aa*, MIP-1α, IL-1β and BL has been supported by clinical studies showing elevated MIP-1α and IL-1β in saliva of *Aa*-positive subjects prior to BL [20], [22].

Ideally cytokines should be studied in a time and site dependent manner referencing levels found at that site and at that time in the context of developing disease. Since cytokine networks are complex, interactive, continuously changing and have redundant functionality, interpretation of levels of cytokines at one point in time are fraught with errors [26]. Our assessments have taken these issues into account by evaluating a site-specific disease in the context of a subject in a longitudinal model. However, we recognize the vulnerability of our interpretation based on the fact that the time between our measurements was 6-months. We propose to use 3-month intervals in future studies; however, it seems reasonable to assume that calcium release from collagen in bone remodeling could take as long as 6–9 months to be seen radiographically [19]. Nevertheless, with these shortcomings in mind, this is the first report to implicate MIP-1α as a predictive risk biomarker with high specificity and sensitivity in both saliva and GCF at a specific site developing periodontal BL in a longitudinal cohort study.

In this study, cytokines such as IL-8, MIP-1α, and MIP-1β, were significantly elevated in whole saliva in LAP subjects prior to BL. These cytokines along with IL-1β are known to recruit and activate, PMNs, monocytes and macrophages and are consistent with an aggressive acute inflammatory response [24], [27]. In addition, IL-17 and TNF-α were elevated in the saliva of the LAP subjects prior to BL. These cytokines as well as MIP-1α emerged from sites in LAP subjects and have also been associated with BL and thus could serve as diagnostic biomarkers for BL [21]. Saliva is known to collect GCF that emigrates from all sites in the dentition and thus saliva is a repository for both subject and site related material [8]. While saliva from the LAP subjects that were undergoing BL showed a significant overall shift toward markers of acute inflammation, healthy subjects who remained healthy, harbor a health related microflora [23] and showed elevated levels of chronic inflammatory markers in their saliva. Several other cytokines such as IL-4, TNF-β and MCP-3 as well as IL-10 and IL-13 were also elevated in saliva of healthy subjects (Fig. 4 and Fig. S1). These cytokines have been implicated in healing and response to injury [27]. In this case it is possible that the host cytokines controlled disease progression [28].

Of particular interest were the distinct differences seen when the four sites from the same LAP subject who developed BL were analyzed because only one of the four sites showed BL while the others remained healthy. In this case the one BL site prior to BL, unlike any of the other sites, showed elevated MIP-1α levels. It is noteworthy that sites that remained healthy showed IL-6 levels that were significantly elevated in these same LAP subjects that developed disease at another site. In a second analysis IL-8 showed elevated levels in LAP sites that remained healthy at the time disease was detected at another site. The relationship of IL-6 and IL-8 to MIP-1α needs to be better understood.

Previously we showed that subgingival sites harbored a microbial consortium consisting of *Aa*, *Filifator alocis* and *Streptococcus parasanguinis* 6–9 months prior to BL, while the other three sites that remained healthy in the same individual showed a health related flora [23]. These findings suggest that the consortium that colonized BL sites could be responsible for affecting the host response at that site (bacterial colonization precedes the host response). These LAP consortium microbes are known to be capable of suppressing immune responsiveness while *Aa*/LPS could activate epithelial cells or monocytes in the underlying connective tissue and thus could be responsible for elevated MIP-1α [29]. Therefore, as suggested by our statistical model and our microbiological data, the local site appears to be more relevant as a predictor of BL than subject derived data (see Tables 6 and 7) [23].

Several questions remain unanswered. Can we assume that the microbial shift causes the local cytokine response? Alternatively is it possible that the local cytokine reaction is responsible for the subsequent microbial shift? It seems likely that the leading edge of the massive subgingival bacterial front challenges the thinning pocket epithelial lining that lies in direct contact with the adjacent highly vascular connective tissue. Chemokines and cytokines secreted by the defending epithelial barrier produce innate response elements that are likely intended to induce a homeostatic balance [24]. While the presumed goal of this response is protection against disease, the specifics of these relationships still need to be proven in vivo [30]. Understanding these complex interactions could provide us with a better understanding of pathogenesis of periodontitis and other mucosally initiated infections.

With this data in hand a strategy should be considered to test MIP-1α as well as other potential inflammatory cytokines such as TNF-α and IL-17 as risk markers for BL on both a site and subject level in long term clinical studies. Using these tools, diagnosis of patients and sites at risk for disease can be improved which can lead to better more cost effective methods of prevention and treatment in this pandemic mucosal infectious disease.

## Supporting Information

Figure S1 **Salivary cytokines from healthy and bone loss subjects: Salivary cytokines depressed at the time bone loss was detected.** Saliva from healthy subjects was compared to subjects who developed bone loss. The cytokines that showed significant differences are illustrated 6 months prior to bone loss and compared to levels at the time bone loss was detected (labeled disease) and to salivary levels found in subjects who started healthy and remained healthy. Letters that are different (A vs B) are significantly different at the p<0.05 level. IL-13, IL-6, IL-7 and IL-10 all show lower levels at time disease was detected.
(TIF)

Figure S2 **Salivary cytokines from healthy and bone loss subjects: Other salivary cytokines depressed at the time bone loss was detected.** Saliva from healthy subjects was compared to subjects who developed bone loss. The cytokines that showed significant differences are illustrated 6 months prior to bone loss and compared to levels at the time bone loss was detected (labeled disease) and to salivary levels found in subjects who started healthy and remained healthy. Letters that are different (A vs B) are significantly different at the p<0.05 level. IL-2 and IFN-γ are lower at time disease was detected as compared to health and prior to disease detection.
(TIF)
