# Supplementary figures and images for: Macrophage Inflammatory Protein-1α Shows Predictive Value as a Risk Marker for Subjects and Sites Vulnerable to Bone Loss in a Longitudinal Model of Aggressive Periodontitis
Source: PLoS One. 2014 Jun 5;9(6):e98541. doi: 10.1371/journal.pone.0098541 (PMC4047026; doi:10.1371/journal.pone.0098541)

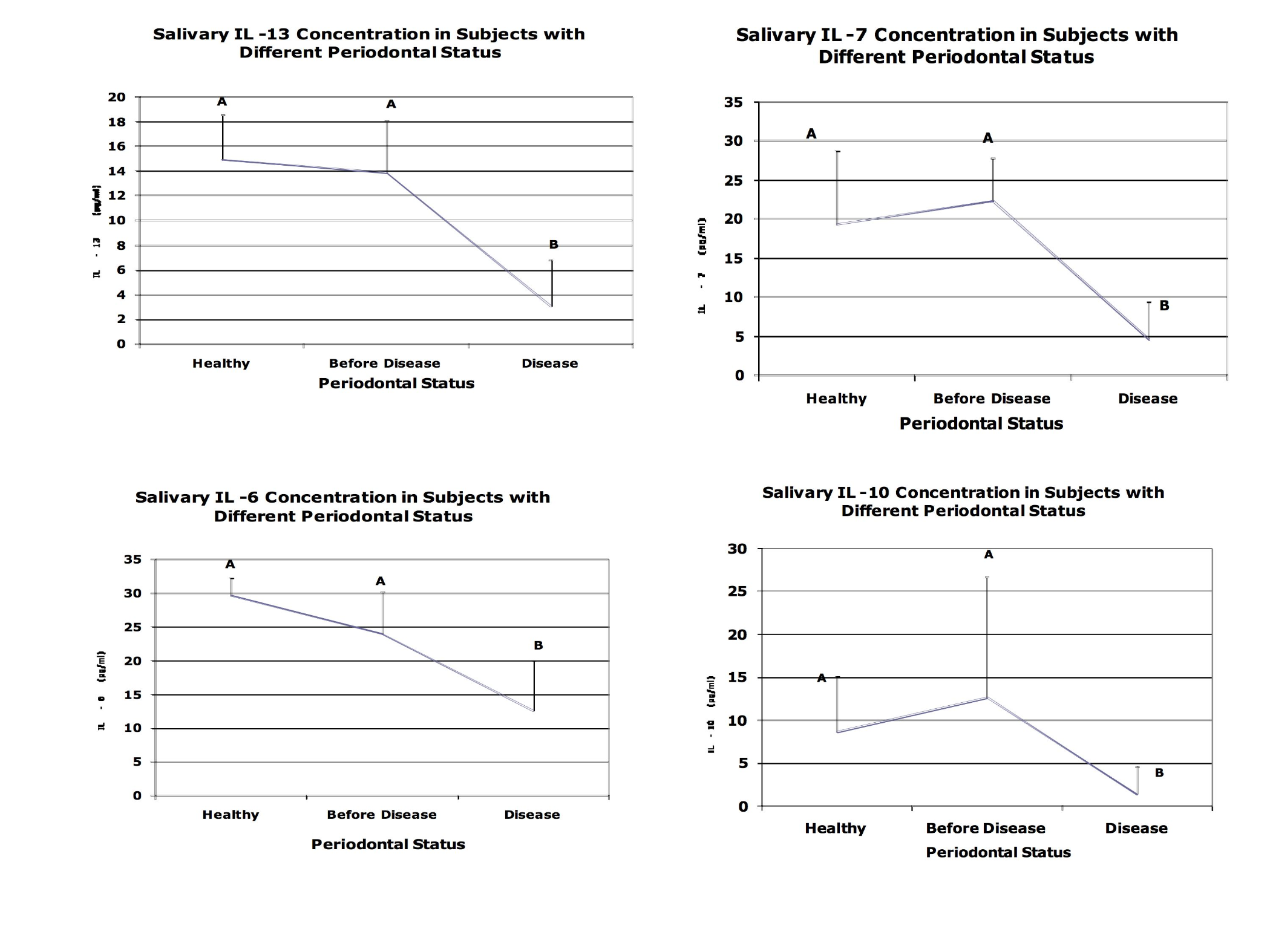

Supplement: Figure S1 — Salivary cytokines from healthy and bone loss subjects: Salivary cytokines depressed at the time bone loss was detected. Saliva from healthy subjects was compared to subjects who developed bone loss. The cytokines that showed significant differences are illustrated 6 months prior to bone loss and compared to levels at the time bone loss was detected (labeled disease) and to salivary levels found in subjects who started healthy and remained healthy. Letters that are different (A vs B) are significantly different at the p<0.05 level. IL-13, IL-6, IL-7 and IL-10 all show lower levels at time disease was detected. (TIF) [file pone.0098541.s001.tif]

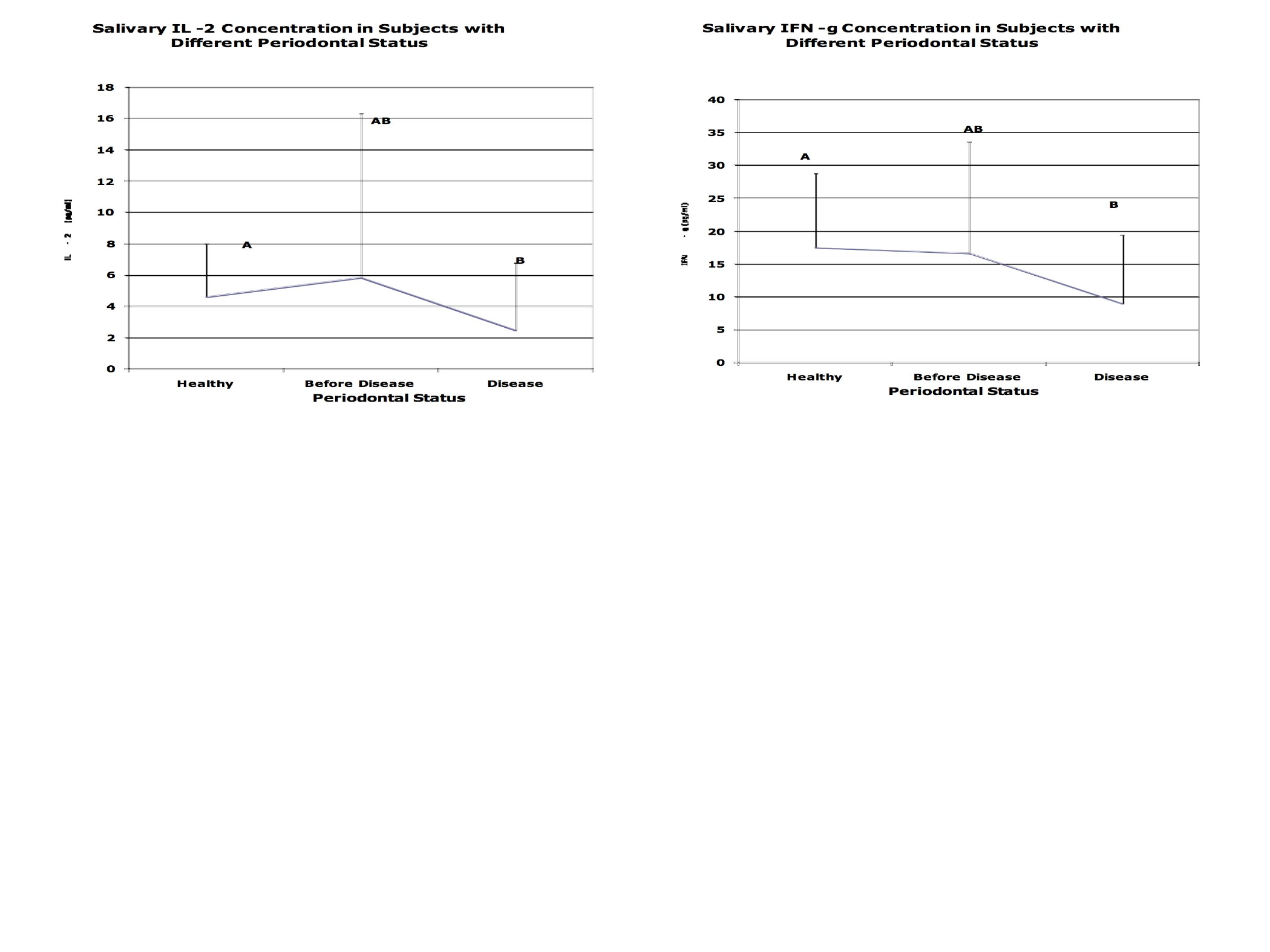

Supplement: Figure S2 — Salivary cytokines from healthy and bone loss subjects: Other salivary cytokines depressed at the time bone loss was detected. Saliva from healthy subjects was compared to subjects who developed bone loss. The cytokines that showed significant differences are illustrated 6 months prior to bone loss and compared to levels at the time bone loss was detected (labeled disease) and to salivary levels found in subjects who started healthy and remained healthy. Letters that are different (A vs B) are significantly different at the p<0.05 level. IL-2 and IFN-γ are lower at time disease was detected as compared to health and prior to disease detection. (TIF) [file pone.0098541.s002.tif]
